# Computational screen and experimental validation of anti-influenza effects of quercetin and chlorogenic acid from traditional Chinese medicine

**DOI:** 10.1038/srep19095

**Published:** 2016-01-12

**Authors:** Zekun Liu, Junpeng Zhao, Weichen Li, Li Shen, Shengbo Huang, Jingjing Tang, Jie Duan, Fang Fang, Yuelong Huang, Haiyan Chang, Ze Chen, Ran Zhang

**Affiliations:** 1 Medical College, Hunan Normal University, Changsha, Hunan 410013, China; 2 College of Life Sciences, Hunan Normal University, Changsha, Hunan 410013, China

## Abstract

The Influenza A virus is a great threat for human health, while various subtypes of the virus made it difficult to develop drugs. With the development of state-of-art computational chemistry, computational molecular docking could serve as a virtual screen of potential leading compound. In this study, we performed molecular docking for influenza A H1N1 (A/PR/8/34) with small molecules such as quercetin and chlorogenic acid, which were derived from traditional Chinese medicine. The results showed that these small molecules have strong binding abilities with neuraminidase from H1N1 (A/PR/8/34). Further details showed that the structural features of the molecules might be helpful for further drug design and development. The experiments *in vitro*, *in vivo* have validated the anti-influenza effect of quercetin and chlorogenic acid, which indicating comparable protection effects as zanamivir. Taken together, it was proposed that chlorogenic acid and quercetin could be employed as the effective lead compounds for anti-influenza A H1N1.

Influenza A virus is a type of orthomyxoviridae virus which is highly implicated in human health^1^. Through infecting the mucosa of upper respiratory tract, the influenza A virus could induce the acute respiratory disease^1^.Previously, a large number of biological studies have characterized the molecular basis of influenza A virus. Although there were a number of distinct types, in all influenza A viruses, 8 genes were conservatively encoded by RNA segments and could be translated into 11 different proteins through distinct open reading frames (ORFs)^2^.The classification of different influenza A virus subtypes were based on the two surface glycoproteins including hemagglutinin (HA) and neuraminidase (NA)^3^. Previously, it was identified that NA was critical for the replication and spread of influenza A virus, and NA inhibitor could serve as the anti-influenza A drugs^4^,^5^. These understandings provide great help to further biological and medical studies of influenza A.

Since the epidemic influenza A is threat to public health, it is critical to develop anti-influenza A drug. Currently, there are three anti-influenza A drugs including amantadine, oseltamivir and zanamivir^6^. Amantadine is the inhibitor of the matrix protein M2, while oseltamivir and zanamivir are inhibitors of neuraminidase (NA)^6^. Although these drugs are effective, the drug-resistant strains of influenza A virus also emerged with the wide usage of these drugs. So, it is critical to develop new anti-influenza A drug. Previously, abundant traditional Chinese medicines (TCMs) from herbaceous plants such as *Forsythia suspense*, *Mangifera indica*, *Hypericum perforatum* and *Chaenomeles speciosa*, were proved to be effective in clinical practice while the complication of their composition remains to be discovered^7^,^8^,^9^,^10^. With the advancement of the bioanalysis technology, a number of small molecules such as quercetin, chlorogenic acid, hypericin, rosmarinci acid and chrysophanol derived from the TCMs were found to be bioactive^11^,^12^,^13^,^14^. For example, as the most frequently studied flavonoid, quercetin was found to be good for heath through protecting low-density lipoprotein from oxidation^15^, antithrombic effects and antiviral properties, while recent studies also showed quercetin was anti-inflammatory and helpful for reduce the risk of pancreatic cancer for smokers^16^,^17^. Chlorogenic acid, a natural polyphenol product from various plants, has antiviral and antihypertensive activity, and could protect dopaminergic neuron from neuroinflammation^18^,^19^,^20^.

In this study, we employed the virtual screen method to explore the potential anti-influenza A activity of several TCM small molecules including quercetin and chlorogenic acid. The H1N1 (A/PR/8/34) subtype was employed for computational and experimental studies. Autodock was used to perform the molecular docking, and the structure of NA protein was modeled from previously reported N1 structure. The results showed that, it was found that the computed binding energy between NA and the two molecules were comparable with the results that from zanamivir. To test the antiviral ability of quercetin and chlorogenic acid, the experiments *in vitro* and *in vivo* were performed. The results suggested quercetin and chlorogenic acid have the antiviral ability. Further detailed analyses showed that these two molecules could serve as a good start for NA inhibitor-like anti-influenza A drug development.

## Results

### Preparation the structures of A/PR/8/34 H1N1 NA and small molecules

**Figure 1:**
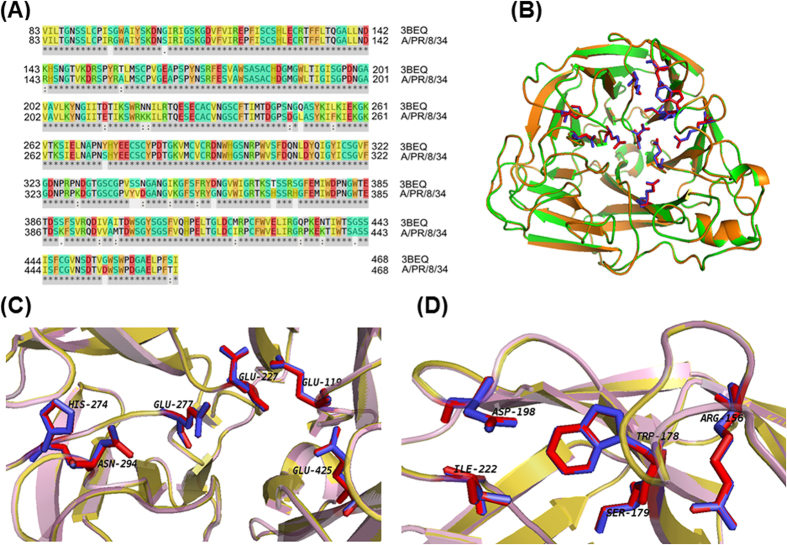
The sequence and structural alignment for the NA from A/PR/8/34 H1N1 and A/Brevig Mission/1/1918 H1N1. (**A**) The sequence alignment result. (**B**) The structural alignment result. The details for structural alignment were presented in (**C**,**D**).

In this study, we selected the structure of NA from A/Brevig Mission/1/1918 H1N1 (PDB ID: 3BEQ) as the template^21^. Through sequence alignment with Clustal Omega^22^, the identity of the two sequences is computed as 93.25%, and the detailed alignment result was presented in Fig. 1A. Previously, it was found that a number of residues including Glu119, Arg156,Trp178, Ser179, Asp/Asn198, Ile222, Glu227, His274, Glu277, Asn294, and Glu425 were critical for the activity of NA^21^. It was observed that these key residues were conserved between the two sequences (Fig. 1A).

To further evaluate the modeling, the modeled structure was compared with the template, and the result was presented by PyMOL^23^. It was obvious that the two structures were nearly identical (Fig. 1B). The detailed results for the local structure of the key residues were presented in Fig. 1C,D, which indicated that the pocket for NA activity is structurally conserved. Taken together, it was observed that the modeled structure is reliable for further computational studies.

### Molecular docking between NA and small molecules

**Figure 2:**
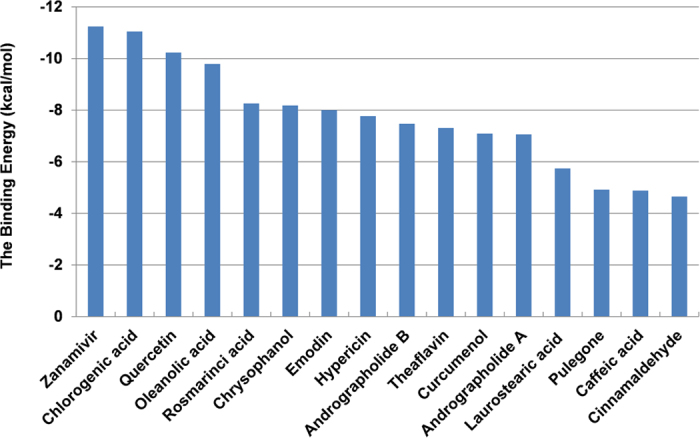
The top15 small molecules with high binding free energies with NA, while zanamvir served as the control.

To further investigate the potential binding between NA and the compounds, the molecular docking was performed. The molecular docking between zanamvir and NA was employed as the control to evaluate the binding ability of other molecules. The binding energies for the fifteen small molecules from docking results were summarized in Fig. 2, which showed that quercetin and chlorogenic acid had highest binding energies comparable with zanamvir. Thus, further investigations in this study were focused on quercetin and chlorogenic acid. The detailed docking results such as binding energies and inhibition constants were presented in Table 1. It was observed that the binding energies between NA and the two molecules are comparable with zanamvir. The docking energy for zanamvir, quercetin and chlorogenic acid were −11.24 kcal/mol, −10.23 kcal/mol and −11.05 kcal/mol, respectively. These results indicated that the NA binding ability of the small molecules were useful for further studies.

To further dissect the docking energies, the contributions of different energies were analyzed. For the interaction between NA and small molecules, the van der Waals’ interaction, hydrogen bond (H-bond), desolvation energy and electrostatic energy contributed as the major part (vdW + Hbond + desolv). In the docking results, it was observed that the vdW + Hbond + desolv energy for chlorogenic acid is −11.1 kcal/mol, which is equal with zanamvir. For quercetin, the result was smaller as −8.09 kcal/mol, respectively. Zanamvir aslo has the highest electrostatic energy as −3.1 kcal/mol, while the electrostatic energies for quercetin and chlorogenic acid were −0.59 kcal/mol and −2.49 kcal/mol, respectively. The internal energy and inhibition constant were also calculated and presented in Table 1, while all the results indicated these small molecules have the potential to binding NA. Furthermore, the inhibition constants calculated for zanamivir, quercetin and chlorogenic acid were 5.73, 31.97 nd 7.98nM, respectively (Table 1), which indicated that quercetin and chlorogenic acid might have considerable NA inhibition ability.

**Figure 3:**
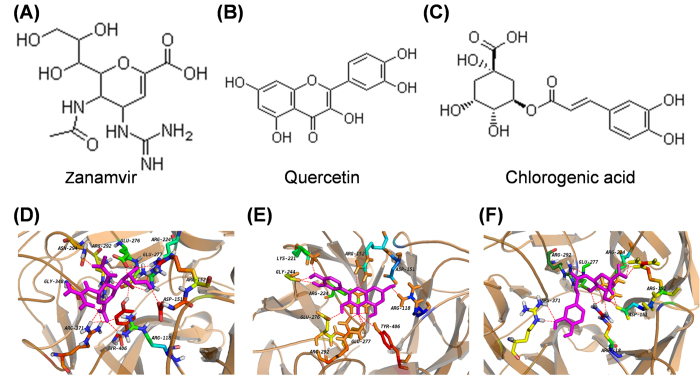
The structures of zanamvir (**A**), quercetin (**B**), chlorogenic acid (**C**), and the details of the binding between NA and zanamvir (**D**), quercetin (**E**), chlorogenic acid (**F**).

To further evaluate the druggabilities, toxicities and promiscuities of quercetin and chlorogenic acid, FAF-Drugs3^24^ was employed to perform chemical informatic analyses for zanamvir, quercetin and chlorogenic acid. The structures of the molecules were presented in Fig. 3, while the results were showed in Table 2. For quercetin and chlorogenic acid, it was observed that: the molecular weights were similar as ~500; the logarithm of the partition coefficient between water and 1-octanol (known as log*P*) was less than five; the number of rotatable bonds is less than 10; the number of hydrogen atoms acceptors is less than 10; the number of hydrogen atoms donors is less than 10. Thus, quercetin and chlorogenic acid matched Lipinski’s rule of five^25^,^26^, which indicated that these two compounds had low toxicities and promiscuities, and high druglikeness.

### Structural insights of the docking complex

To further understanding the details of NA binding for the molecules, the molecular structures for the NA in complex with the molecules were analyzed. The complex structures for zanamvir, quercetin and chlorogenic acid were presented in Fig. 3. The key residues were labeled while the interaction details were visualized. As the important molecular mechanism, the H-bond were analyzed and shown as dotted line, while the residues from NA for H-bond were summarized in Table 3.

**Figure 4:**
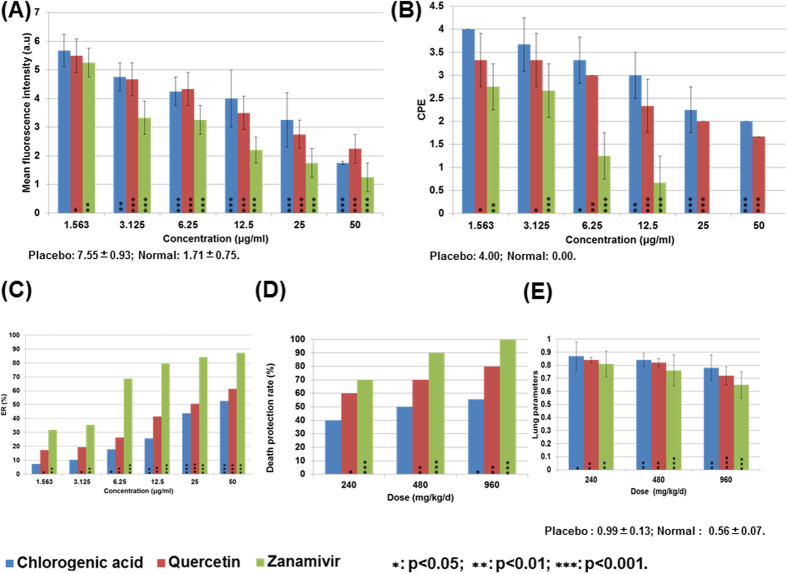
The summary of the experimental results for cytopathic effect (CPE) (**A**), mean fluorescence intensity (**B**), effect rate (**C**), death protection rate (**D**) and lung index (**E**).

As the result presented in Fig. 3D, it was observed that there were 21 H-bonds between zanamvir and 11 residues of NA. The binding area was critical for the activity of NA, while the abundant H-bonds indicated that the H-bonds were important for the binding between small molecules and NA. Figure 4E shows that the binding of quercetin could generate 17 H-bonds, which indicated that quercetin could serve as a leading molecule as NA inhibitor. For chlorogenic acid, 15 H-bonds could be formed with NA (Fig. 4F), while the average energy for H-bonds was higher than zanamvir.

### Validation of NA inhibition ability for small molecules

To validate the NA inhibition ability for small molecules that showed high binding potential in the docking experiments, experiments including *in vitro* neuraminidase activity assay and cytopathic effect assay were carried out. Firstly, MTT cell proliferation assay was performed to obtain the maximum non-toxic dose (MNLD). It was observed that the MNLDs for zanamvir, quercetin and chlorogenic acid were 71.31, 61.54 and 73.72 μg/ml, respectively, which indicated that the toxicities of the three compounds might be similar. Then a series of concentrations including 1.56, 3.13, 6.25, 12.5, 25 and 50 μg/ml were employed, while the *in vitro* neuraminidase activity assay and cytopathic effect assay were parallel performed. From the results shown in Table 4 (Fig. 4A), it was observed that the three compounds could significantly inhibit the activity of neuraminidase at the concentrations of 1.56 μg/ml for zanamvir and quercetin and 3.13 μg/ml for chlorogenic acid (Fig. 4A). Furthermore, since the fluorescence intensity was reported as no significant difference in comparison with the control, it seems that the activity of neuraminidase was nearly fully inhibited by quercetin and chlorogenic acid under the concentration of 50 μg/ml. These results indicated that quercetin and chlorogenic acid showed considerably comparable neuraminidase inhibition abilities.

Furthermore, the protection effect of the three compounds for cells were evaluated and compared. The cytopathic effect (CPE) and protective effect rate (ER) were calculated and presented in Table 4 and Fig. 4B,C. It was observed that the cytopathic effect (CPE) and protective effect rate (ER) for cells treated with quercetin and chlorogenic acid were significantly low and high, respectively, which indicated that the two compounds have protection effect for influenza-infected cells. Interestingly, the CPE results were consistent with neuraminidase activity assay that the quercetin and chlorogenic acid were effective at low concentrations of 1.56 μg/ml and 3.13 μg/ml, respectively. The MTT based protective effect rate (ER) assay for the three compounds showed a dose-dependent results (Table 4, Fig. 4C).

### *In vivo* validation of anti-influenza abilities of quercetin and chlorogenic acid

Since the infection of influenza could cause the death, increase lung index and pneumonia, survival rate and lung index (Lung weight / Body weight) could be employed to evaluate the protection effect of the compounds^27^. In this study, influenza A H1N1 (A/PR/8/34) infected mice were treated with compounds of different doses including 240, 480 and 960 mg/kg/d through intragastric administration. As shown in Table 5 and Fig. 4D, treatments with chlorogenic acid and quercetin at the dose of 240 mg/kg/d could promote the survival rates from 40% and 60% to 56% and 80%, respectively. Furthermore, the inhibition rate of lung index were measured for the chlorogenic acid and quercetin treated mice as 20.2%, 14.4%, 12.2%, and 26.1%, 16.5%, 14.7% at the doses of 240, 480 and 960 mg/kg/d. It was observed that the inhibition rates were in a dose-dependent manner, while the survival rate and lung index for quercetin (80%, 0.72 ± 0.07) at 960 mg/kg/d were similar with zanamvir at 480 mg/kg/d (90%, 0.65 ± 0.10) (Table 5 and Fig. 4E).

## Material and methods

### Molecular structure preparing

The NA structure of A/PR/8/34 H1N1 was modeled from the structure of A/Brevig Mission/1/1918 H1N1 (PDB ID: 3BEQ)^21^ by SWISS-MODEL Workspace^28^,^29^,^30^. The identity of two NA sequences between A/Brevig Mission/1/1918 and A/PR/8/34 was 93.25%, which indicated that the modeled structure was reliable. All the visualizations of molecular structures were performed with PyMOL (Version 1.7.2, available at http://pymol.org)^23^. To find potential anti-influenza small molecules, we curated the plants from traditional Chinese medicine which were identified to have anti-influenza effects from published literature in Chinese. Then the components which were bioactive and identified in these plants were collected. In total, fifteen small molecules were collected for molecular docking inlcuding hypericin from *Forsythia suspense*, chlorogenic acid, laurostearic acid, oleanolic acid, caffeic acid, quercetin, theaflavin from *Honeysuckle*, andrographolide A, andrographolide B, curcumenol from *Andrographis paniculata*, chrysophanol, emodin from *Rheum*, pulegone, rosmarinci acid from Rosmarinus officinalis, and cinnamaldehyde from the root of *Chinese thorowa*. The structures for these small molecules were retrieve from the ZINC database, which is a curated collection of commercially available chemical compounds^31^.

### Molecular docking

In this study, AutoDock software version 4.2 (http://autodock.scripps.edu) were employed to perform molecular docking as previously described^32^. AutoDock incorporates limited flexibility in the receptor, it combines an empirical free energy force field with a Lamarckian Genetic Algorithm, providing fast prediction of bound conformations with predicted free energies of association^33^,^34^. The binding energy is calcualted as the sum of the intermolecular energy and the torsional free-energy^34^,^35^,^36^. The modeled NA structure was employed as the receptor to docking with these small molecules. The AutoDockTools1.5.6 software was employed to prepare the docking procedures^33^. The default 0.375 Å spacing was adopted for the grid box, and the volume was set as 72 × 72 × 72. The key residues for NA were selected as the docking pocket, which contains eight residues including Arg118, Asp151,Arg152, Arg224, Glu276, Arg292, Arg371, Tyr40^21^,^37^,^38^,^39^.The number of runs was set as 100. Furthermore, FAF-Drugs3 software (http://fafdrugs3.mti.univ-paris-diderot.fr) was used to carried out chemical informatics analyses for the small molecules^24^.

### Virus strain, cell culture and animals

Animals (BALB/c mice) were used according to the guidelines of the National Institutes of Health for care and use of laboratory Animals. All experiments requiring animals received prior approval from the Animal Care and Use Committee of the College of Medicine, Hunan Normal University. A/Puerto Rico/8/34(H1N1) virus was prepared as described in our preview research^40^. The influenza A/Puerto Rico/8/34(H1N1) virus and Madin-Darby canine kidney (MDCK) cells were preserved in Molecular Virology Lab, School of Life Sciences, Hunan Normal University. MDCK cells were cultured in Dulbecco’s modified Eagle’s medium (DMEM) supplemented with 10% fetal calf serum (FCS) and incubated in an atmosphere of 5% CO_2_ at 37 °C which had been humidified. Female BALB/c mice (19–22g) were obtained from SJA Laboratory Animal Co., Ltd., Hunan, China.

### Reagents and compounds

Chlorogenic acid (C3878), quercetin (Q4591), dimethyl sulfoxide (DMSO) (276855), sodium carboxymethyl cellulose (CMC) (C5678), zanamivir (102157A) and MTT (45956A) (3-(4,5-Dimethylthiazol-2-yl)-2,5-diphenyltetrazolium bromide) were purchased from Sigma-Aldrich Trading Co., Ltd., Shanghai, China. Zanamivir was provided by Adamas Pharmaceuticals. Molecules were dissolved in DMSO and CMC for *in vitro* and *in vivo* experiments.

### Cytotoxic Assay for Zanamivir, quercetin and chlorogenic acid

The noncytotoxic doses of three kinds of molecules on MDCK cells were determined by the methyl-thiazolyl-tetrazolium (MTT) method respectively and the maximum non-toxic concentrations (TC0) were calculated by R package ‘drc’^41^.

### The cytopathic effect assay

MDCK cells grown in 24-well plates for 24 h were washed with serum-free medium and then infected with 400 μl of 100 TCID_50_ influenza virus A/PR/8/34 for 2 h at 37 °C. Unabsorbed virus was removed and the cells were treated with various doses of chlorogenic acid or quercetin solution (1.56, 3.13, 6.25, 12.5, 25.0, 50.0 μg/ml). The uninfected cell controls and placebo controls were included in each test. Zanamivir served as the positive control drug, which was used for estimating antiviral efficiency. Then, the plates were incubated at 37 °C. This experiment was repeated 3 times. The cell monolayers were examined by microscope every 12 h, and the cytopathic effect (CPE) induced by influenza virus was marked on a scale from 0 (no CPE) to 4 (100% CPE).

### Evaluation of anti-H1N1 effect rate

MDCK cells were cultured in 96-well plates and cultured in minimum essential medium (MEM) for 2 days at 37 °C to >90% confluence. Cells were washed with serum-free Medium and infected with 100 μl of 100 TCID_50_ influenza virus A/PR/8/34 (H1N1) for 2 h at 37 °C. After removing the virus-containing medium, the cells were treated with various doses of chlorogenic acid, quercetin and zanamivir solution in MEM. Zanamivir served as positive control in all experiments, which was estimated for antiviral efficiency. Then, the 96-well microtiter plates were cultured at 37 °C and 5% CO_2_ for 48 h. The antiviral efficiency was determined with MTT method, the absorbance of the solution was measured spectrophotometrically at 570 nm with automated plate reader, while the effect rate (ER) was defined as follows:


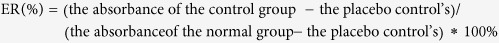


### Enzymatic Assay of NA Activity by Fluorometric Substrate

When the monolayer MDCK cells cultured in 96-wells plates grew to 90% of plates, the maintenance medium was removed from replicates. The wells were washed with serum-free medium and inoculated with 100 μl of 100TCID_50_ influenza virus A/PR/8/34 (H1N1) for 2 h at 37 °C incubator which contained 5% CO_2_. The unabsorbed virus was removed, the cells were treated with molecules solution in a series of concentration from 1.563 ug/ml to 50 μg/ml. Zanamivir was used to evaluate the ability of molecules antiviral activity and served as the positive control. Then, the plates were sealed and incubated at 37 °C for 48 h. The cells were frozen and thawed three times. 8 min later, the supernatant of the cells was harvested for the assay of neuraminidase by Neuraminidase Inhibitors Identification Kit P0306-1.

### *In vivo* anti-influenza virus experiments

A total number of 198 BALB/c female mice were randomly divided into 11 groups. All of mice were anesthetized by 1% pentobarbital sodium solution, and then infected with 24MLD_50_ mouse adapted influenza virus A/PR/8/34 suspension with 0.1% bovine serum albumin (BSA) in PBS through intranasal administration.

The oral administrations of chlorogenic acid and quercetin were suspended in 0.1% (w/v) sodium carboxymethylcellulose (CMC) solution, and 24 h after virus infection the suspension was delivered to the mouse with several doses of chlorogenic acid and quercetin solution (240, 480, 960 mg/kg/d) twice daily for 4d. Zanamvir and CMC were given as a positive controlled and placebo controlled, respectively.

Ten mice per group were used to observe for clinical signs of infection or death in 14d. On day 5 of infection with virus, lung infection parameters were assessed and per group had 8 animals. Mice were weighed and sacrificed, and then their lungs were removed and weighted. Body weight and lung weight were assigned to A and B, lung index of the drug-treated group and lung index of the infected controls were assigned to C and D, respectively. The lung index means B/A × 100%, the lung index inhibition means (D – C)/D × 100%.

## Discussion

Recently, influenza A became a great threat to human health, while the effective drugs were limited because of the resistance to drugs generated from various mutation^6^,^29^,^42^,^43^,^44^,^45^. However, conventional drug development is time-consuming and labor-intensive, while computer-aid drug design could provide helpful information. In this study, computational screen was employed to discover small molecules with NA inhibition ablities and anti-influenza potential from TCMs. Since the protein structure is the basis for molecular docking based screening, the structure of NA (A/PR/8/34 H1N1) was computationally modeled from NA (A/Brevig Mission/1/1918 H1N1) through state-of-art homology-based computation. Through computational screen, we proposed that small molecules from TCMs including chlorogenic acid and quercetin might be highly potential anti-influenza candidate. The *in vitro* and *in vivo* experiments validated the protective effects of the two molecules. It was observed that chlorogenic acid and quercetin could protect influenza A H1N1 infected cells and mice at a comparable level of zanamvir, while quercetin acts at a lower dose. In this study, the small molecules were delivered through intragastric administration, which ensured the absorption. However, the rate of oral absorption of zanamvir is limited as 2%^10^, which indicated that the deliver efficiency of chlorogenic acid and quercetin should be carefully investigated. Furthermore, the *in vivo* absorption and metabolism of small molecules is complicated. Previous study showed that the metabolism product of oseltamivir carboxylate for oseltamivir is the active component with anti-influenza activity^46^. For quercetin, previous study showed that it was absorbed at the rate of 59.1% after hydrolyzing in intestinal tract^47^, while only 5.3% was absorbed directly^48^. The absorption of chlorogenic acid was also low and relied on methylation in intestinal tract^49^,^50^. Thus, further studies should be contributed to analyze the pharmacokinetics for these two small molecules. Taken together, the molecular docking results indicated that chlorogenic acid and quercetin could serve as leading molecules for the development of anti-influenza A drug, while this study provide an initial step for the further design and improvement.

## Additional Information

**How to cite this article**: Liu, Z. *et al.* Computational screen and experimental validation of anti-influenza effects of quercetin and chlorogenic acid from traditional Chinese medicine. *Sci. Rep.* **6**, 19095; doi: 10.1038/srep19095 (2016).

## Figures and Tables

**Table 1:** The energies for the binding between the small molecules and NA.

Molecules	(vdW + Hbond + desolv) energy(kcal/mol)	Electrostatic energy (kcal/mol)	Total Internal energy (kcal/mol)	The best docking energy (kcal/mol)	Inhibition constant (nM)
Zanamvir	−11.1	−3.1	−17.92	−11.24	5.73
Quercetin	−8.09	−0.59	−19.65	−10.23	31.97
Chlorogenic acid	−11.1	−2.49	−22.43	−11.05	7.98

**Table 2:** The chemical informatics analyses of zanamvir, quercetin and chlorogenic acid.

Molecules	MW	LogP	tPSA	Rotatable bonds	HB donors	HB acceptors	Oral Bioavailability (VEBER)	Oral Bioavailability (EGAN)
Zanamvir	333.32	−2.98	202.79	7	9	11		
Quercetin	302.24	2.17	134.2	1	5	7	Good	Good
Chlorogenic acid	354.31	−0.42	167.6	5	5	9	Good	Good

**Table 3:** The residues for the H-bonds between the small molecules and NA.

Molecules	H-bond residues
Zanamvir	ARG118, ASP151, ARG152, ARG224, GLU276, GLU277, ARG292, ASN294, GLY348, ARG371, ARG406
Quercetin	ARG118, ASP151, ARG152, LYS211, ARG224, GLY244, GLU276, GLU277, ARG292, TYR406
Chlorogenic acid	ARG118, ASP151, ARG152, ARG224, GLU277, ARG292, ARG371

**Table 4:** Validation of NA inhibition ability for small molecules.

	Concentration (μg/ml)	Mean CPE ± S.D.	ER(%)	Mean fluorescence intensity (A.U ± S.D.)
Chlorogenic acid	50	2.00 ± 0.00***	52.7***	2.25 ± 0.39***
	25	2.25 ± 0.50***	43.8***	3.31 ± 0.25***
	12.5	3.00 ± 0.50**	25.6**	4.11 ± 0.32***
	6.25	3.33 ± 0.50*	17.8*	4.43 ± 0.38***
	3.125	3.67 ± 0.58	10.2	4.75 ± 0.45**
	1.563	4.00 ± 0.00	5.3	5.67 ± 0.57
Quercetin	50	1.67 ± 0.00***	59.3***	1.74 ± 0.16***
	25	2.00 ± 0.00***	50.6***	2.67 ± 0.20***
	12.5	2.33 ± 0.58***	41.5**	3.50 ± 0.26***
	6.25	3.00 ± 0.00**	26.3**	4.34 ± 0.31***
	3.125	3.33 ± 0.58*	19.4*	4.63 ± 0.41***
	1.563	3.33 ± 0.58*	17.7*	5.48 ± 0.52*
Zanamvir	50	0.00 ± 0.00***	87.3***	1.24 ± 0.11***
	25	0.00 ± 0.00***	84.1***	1.73 ± 0.15***
	12.5	0.67 ± 0.58***	79.6***	2.20 ± 0.25***
	6.25	1.25 ± 0.50***	68.8***	3.27 ± 0.29***
	3.125	2.67 ± 0.58***	35.3**	3.34 ± 0.40***
	1.563	2.75 ± 0.50**	31.7**	5.35 ± 0.48**
Placebo	–	4.00 ± 0.00	–	7.54 ± 0.61
Normal	–	0.00 ± 0.00	–	1.71 ± 0.14

*p < 0.05, **p < 0.01, ***p < 0.001, the values were compared with placebo controls.

**Table 5:** *In vivo* validation of anti-influenza abilities of quercetin and chlorogenic acid.

Treatment	Dose (mg/kg/d)	Survivors/totals (%)	Mean lung parameters	
			Lung Index ± S.D. (%)	Lung indexInhibition %
Zanamivir	960	10/10 (100)***	0.65 ± 0.10***	34.0
	480	9/10 (90)***	0.74 ± 0.11***	25.3
	240	7/10 (70)***	0.81 ± 0.11**	18.3
Quercetin	960	8/10 (80)**	0.73 ± 0.07***	26.3
	480	7/10 (70)**	0.82 ± 0.03**	16.5
	240	6/10 (60)*	0.84 ± 0.02**	14.7
Chlorogenic acid	960	5/9 (55.6)*	0.79 ± 0.10**	20.2
	480	5/10 (50)	0.84 ± 0.05**	14.4
	240	4/10 (40)	0.87 ± 0.11*	12.2
Placebo	—	1/10 (10)	0.99 ± 0.13	—
Normal	—	10/10 (100)	0.56 ± 0.07	—

*p < 0.05, ** *p* < 0.01, ***p < 0.001, the values were compared with placebo controls.
